# Glycosylated Hemoglobin (HbA1C) as a Predictor of Early Postoperative Outcomes After Coronary Artery Bypass Grafting: A Single-Center Observational Study

**DOI:** 10.7759/cureus.65567

**Published:** 2024-07-28

**Authors:** Fahad M Alshair, Abdullah H Baghaffar, Mazin A Fatani, Anas K Alqahtani, Abdullah K Al Assiri, Badr M Alsulymani, Abdullah M Sanedi, Saud M Bamousa

**Affiliations:** 1 Cardiac Surgery, King Abdulaziz University Hospital, Jeddah, SAU; 2 Medicine, King Abdulaziz University, Jeddah, SAU; 3 Medicine, University of Jeddah, Jeddah, SAU

**Keywords:** complications, coronary artery bypass grafting, hba1c, glycosylated hemoglobin, diabetes mellitus

## Abstract

Background

Diabetic patients present a majority of patients undergoing surgical revascularization. Hyperglycemia is associated with increased adverse events. Glycosylated hemoglobin (HbA1c) is an effective biological marker for long-term glycemic control. As a result, there is an increased trend in its use as a predictor of adverse outcomes. This study aims to assess the impact of elevated HbA1c on the occurrence of postoperative complications after coronary artery bypass grafting (CABG).

Methods

We conducted a retrospective review of medical records from January 2015 to December 2022 for adult patients who underwent isolated CABG. We assessed patient demographics, medication, laboratory results, HbA1c results, and clinical data. The separate statistical models were designed to assess the predictors for the development of postoperative complications.

Results

This retrospective single-center study was conducted on 289 consecutive adult patients who underwent on-pump CABG. Patient demographics showed that uncontrolled HbA1c was more in females (p=0.022), and hemodialysis patients (p=0.018). Across different levels of HbA1C, there were no significant differences in terms of the incidence of postoperative complications (p=0.788 for infection, p=0.372 for the need for blood transfusion, p=0.721 for heart failure, p=0.692 for arrhythmia, and p=0.712 for death). HbA1c had no predictive value for postoperative complications as indicated by multivariate and stepwise analysis in a separate model for each complication with receiver operator characteristics curves of each model showing similar strength of both multivariate and stepwise models.

Conclusions

In our data, elevated preoperative HbA1c had no predictive value for early complications and intermediate postoperative outcomes. We recommend that surgery should proceed without delay, even if patients have elevated HbA1C levels. As for elective patients with low-risk features and anatomy, optimizing preoperative glycemic control can be considered.

## Introduction

For patients undergoing cardiac surgery, a possibility is present to optimize their preoperative condition in the perioperative period to aid, enhance, and improve postoperative outcomes. These optimizations can be done throughout their operative journey.

One of the most common conditions that patients undergoing cardiac surgery might have and can be optimized in the preoperative period is diabetes mellitus (DM). It is one of the most challenging concerns for public health. According to the latest data from the World Health Organization in 2016, there are an estimated 422 million adults who are affected by DM. This prevalence is estimated to increase and reach 629 million in the year 2045 [1]. When compared to an age and sex-matched population without DM, patients with DM have a two- to four-fold increased risk of developing cardiovascular disease and a two- to five-fold increased mortality due to cardiocerebrovascular disease, accounting for 65% of patient deaths [2].

Patients with diabetes account for roughly 25% of those undergoing coronary revascularization, with coronary artery bypass grafting (CABG) being the recommended method of revascularization for this subset of patients with multivessel coronary artery disease and optimal anatomy [3].

High preoperative blood glucose levels have been clearly linked to a higher incidence of postoperative complications and a lower survival rate following CABG, according to previous investigations. However, these measurements were based on random blood glucose levels, which do not necessarily indicate the long-term state of diabetes control, which may be altered by various concomitant circumstances. Hyperglycemia has been suggested to be a highly significant predictor of mortality and morbidity in patients undergoing cardiac operations who were not previously diagnosed with diabetes [4-6].

One of the most often researched metrics for outcome prediction in cardiac surgery is glycosylated hemoglobin (HbA1c). HbA1c test is a serum blood laboratory test used to measure the efficacy of long-term antidiabetic therapy and control. The development of HbA1c in the bloodstream is performed by a glycosylation reaction that occurs when hemoglobin (Hb) is subjected to free blood glucose, which in hand results in molecular structural changes to Hb. Over the course of an erythrocyte's 120-day lifespan, the rate at which Hb is glycosylated rises in tandem with blood glucose levels. Because the HbA1c test gauges the progress of glycosylation, it offers insight into the blood glucose profile of individuals. The American Diabetes Association (ADA) in the latest 2023 glycemic control guidelines advocates that diabetic subjects should achieve target HbA1c levels of less than 7% because it is associated with a lower risk of diabetes-related complications [7].

Although some articles in the literature argue that high HbA1c levels increase mortality and morbidity, there are studies arguing that there is no relation between them [8-10]. The lack of recent local studies and the contradiction between the results of existing literature, with regard to whether HbA1c can be used as a predictive marker for operative complications in patients undergoing CABG, called our interest to conduct this study alongside other literature to investigate further.

## Materials and methods

Methods

This is a retrospective cohort study that included 289 adult patients who underwent isolated on-pump CABG at King Abdulaziz University Hospital (KAUH) in Jeddah, Saudi Arabia between January 2015 and December 2022. Patients with an available preoperative HbA1c result were included.

The data were collected from the medical records department using an electronic healthcare information system and the laboratory information system after the Institutional Review Board (IRB) approval of the study. Throughout the research process, all ethical considerations were followed. 

We defined uncontrolled levels of HbA1c according to the ADA definition of a target HbA1c level during follow-up of more than 7%. Patients who had levels less than 7% were considered controlled. In addition, complications were collected during the admission of surgery and a year after that during the follow-up period.

Primary and secondary objectives

The primary objectives of this study were 1) to identify the use of uncontrolled levels of HbA1c as a predictor of postoperative outcomes (development of postoperative wound infection, arrhythmia, blood transfusion, low cardiac output syndrome, and mortality) and 2) to compare the outcomes of controlled versus uncontrolled levels of HbA1c on patients undergoing on-pump CABG. The secondary objectives were 1) to evaluate other possible predictors of postoperative complications after on-pump CABG and 2) to identify the prevalence of uncontrolled DM in patients who undergo on-pump CABG.

Statistical analysis

All statistical analyses were performed using the R statistical software, version 4.3.0 (R Foundation for Statistical Computing, Vienna, Austria). We compared the features of adult patients who underwent on-pump CABG and had uncontrolled levels of HbA1c to those of the patients who were controlled preoperatively. We expressed categorical variables as frequencies and percentages, whereas continuous variables were presented as mean ± SD. Statistical differences between patients with controlled and uncontrolled glycemic levels were assessed using a Fisher's exact test or Pearson's Chi-squared test for categorical variables or a Wilcoxon rank sum test for continuous variables.

To identify pre-, intra-, and postoperative risk factors for the development of postoperative complications (mortality, Infection, arrhythmia, heart failure, and need for postoperative packed red blood cell transfusion), univariate analysis using logistic regression was used to identify significant pre- and intraoperative predictors with a p-value less than 0.1. All significant predictors from the univariate analysis were included in a multivariate analysis using logistic regression. To further identify important predictors, stepwise logistic regression analysis was used to exclude non-significant predictors. Every studied postoperative complication was assessed using a separate model. The full multivariate model and stepwise model for each statistical model for each complication were compared using a receiver operator curve. All statistical tests were two-tailed, and p-values <0.05 were considered significant. 

For the survival analysis, the days between the time of surgery and censoring (death or last visit) were used to estimate survival curves (Kaplan-Meier curve) for patients with controlled and uncontrolled levels of HbA1c with the comparison being performed using a log-rank test. To assess the bivariate association between variables and survival time, a univariate Cox-proportional hazard model was performed. Variables with p-values <0.2 were further used as independent variables in an adjusted multivariable Cox regression model. Statistical significance was considered at p<0.05. 

## Results

Patients general characteristics and demographics

The majority were male 245 (84.8%), while 44 (15.2%) were female. The mean age was 58.6 years, and the mean weight was 75.0 kg. Comorbidities included hypertension in 201 (69.6%) patients. 131 (45.3%) patients had controlled levels of HbA1c <7%, whereas 158 (54.7%) patients had uncontrolled levels of >7%. Females had a significantly higher proportion of unsatisfactory glycemic control compared to males (70.5% vs 51.8%, p=0.022). All of the patients who were on hemodialysis had uncontrolled levels of HbA1c (100.0%) (p=0.018). More details about the clinical history of patients are provided in Table 1. 

**Table 1:** Patient characteristics and demographics Fisher's exact test or Pearson's Chi-squared test was used for categorical variables and a Wilcoxon rank sum was used for continuous variables. BMI: body mass index, BSA: body surface area, IHD: ischemic heart disease, DM: diabetes mellitus, HTN: hypertension, DLP: dyslipidemia, PVD: peripheral vascular disease, CKD: chronic kidney disease, Preop: preoperative, COPD: chronic obstructive pulmonary disease

Characteristic	Missing	Overall, N=289	HbA1C <7%, N=131	HbA1C >7%, N=158	p-value
Age in years (mean±SD)	0 (0%)	58.6±10.2	58.0±11.2	59.1±9.3	0.335
Gender, n (%)	0 (0%)				0.022
Male		245 (84.8%)	118 (90.1%)	127 (80.4%)	
Female		44 (15.2%)	13 (9.9%)	31 (19.6%)	
Weight in Kg (mean±SD)	1 (0.3%)	75.0±13.6	75.4±15.4	74.7±11.9	0.853
Height in cm (mean±SD)	0 (0%)	165.7±10.8	165.4±11.9	166.0±9.8	0.640
BMI in kg/m^2^ (mean±SD)	2 (0.7%)	27.0±4.8	26.9±4.2	27.1±5.2	0.867
BSA in m^2 ^(mean±SD)	2 (0.7%)	2.0±1.6	2.0±1.6	2.0±1.6	0.933
HTN, n (%)	0 (0%)				0.775
No		88 (30.4%)	41 (31.3%)	47 (29.7%)	
Yes		201 (69.6%)	90 (68.7%)	111 (70.3%)	
DLP, n (%)	0 (0%)				0.419
No		214 (74.0%)	100 (76.3%)	114 (72.2%)	
Yes		75 (26.0%)	31 (23.7%)	44 (27.8%)	
PVD, n (%)	0 (0%)				>0.999
No		286 (99.0%)	130 (99.2%)	156 (98.7%)	
Yes		3 (1.0%)	1 (0.8%)	2 (1.3%)	
CKD, n (%)	0 (0%)				0.516
No		272 (94.1%)	122 (93.1%)	150 (94.9%)	
Yes		17 (5.9%)	9 (6.9%)	8 (5.1%)	
Preop dialysis, n (%)	0 (0%)				0.018
No		284 (98.3%)	126 (96.2%)	158 (100.0%)	
Yes		5 (1.7%)	0 (0.0%)	5 (3.8%)	
Smoking, n (%)	41 (14%)				0.055
No		193 (77.8%)	84 (72.4%)	109 (82.6%)	
Yes		55 (22.2%)	32 (27.6%)	23 (17.4%)	
COPD, n (%)	0 (0%)				>0.999
No		287 (99.3%)	130 (99.2%)	157 (99.4%)	
Yes		2 (0.7%)	1 (0.8%)	1 (0.6%)	
Asthma, n (%)	0 (0%)				0.332
No		285 (98.6%)	128 (97.7%)	157 (99.4%)	
Yes		4 (1.4%)	3 (2.3%)	1 (0.6%)	
Stroke, n (%)	0 (0%)				0.795
No		277 (95.8%)	126 (96.2%)	151 (95.6%)	
Yes		12 (4.2%)	5 (3.8%)	7 (4.4%)	

Pre-, intra-, and postoperative characteristics

The mean values for key continuous variables were as follows: systolic blood pressure (SBP) at 128.8 mmHg, diastolic blood pressure (DBP) at 72.3 mmHg, heart rate (HR) at 79.8 bpm, Hb at 13.1g/dL, white blood cell (WBC) count at 9.5x10^3^/μL, platelet count at 257.7x10^3^/μL, creatinine at 111.7 μmol/L, troponin at 3.4 ng/mL, EF at 45.5%, number of vessels involved in the procedure at 3.0, CPB time at 115.9 minutes, cross-clamp time at 72.0 minutes, duration of mechanical ventilation at 1.9 days, length of stay in the intensive care unit at 4.0 days, and length of stay in the hospital at 13.6 days. There were no significant differences between patients with poor and good glycemic control in terms of all pre- and intraoperative characteristics (Table 2).

**Table 2:** Pre-, intra-, and postoperative characteristics Fisher's exact test or Pearson's Chi-squared test was used for categorical variables and a Wilcoxon rank sum was used for continuous variables. SBP: systolic blood pressure, DBP: diastolic blood pressure, HR: heart rate, Hb: hemoglobin, WBC: white blood cells, PLT: platelets, PCI: percutaneous coronary intervention, EF: ejection fraction, CPB: cardiopulmonary bypass, ICU: intensive care unit

Characteristic	Missing	Overall, N=289	HbA1C <7%, N=131	HbA1C >7%, N=158	p-value
SBP in mmHg (mean±SD)	1 (0.3%)	128.8±23.2	130.6±23.3	127.2±23.1	0.210
DBP in mmHg (mean±SD)	1 (0.3%)	72.3±15.9	73.1±16.5	71.6±15.4	0.355
HR in BPM (mean±SD)	1 (0.3%)	79.8±17.3	78.2±14.8	81.1±19.1	0.296
Hb in g/dL (mean±SD)	0 (0%)	13.1±2.0	13.1±2.1	13.0±2.0	0.481
WBC in cells×10^9^/L (mean±SD)	0 (0%)	9.5±13.3	10.3±19.4	8.8±3.2	0.555
PLT in cells×10^9^/L (mean±SD)	0 (0%)	257.7±71.2	252.6±64.6	261.9±76.2	0.408
Creatinine in micromoles/L (mean±SD)	0 (0%)	111.7±122.2	125.2±169.4	100.4±57.9	0.828
Troponin in ng/mL (mean±SD)	2 (0.7%)	3.4±9.1	3.4±8.5	3.5±9.7	0.246
PCI, n (%)	0 (0%)				0.345
No		222 (76.8%)	104 (79.4%)	118 (74.7%)	
Yes		67 (23.2%)	27 (20.6%)	40 (25.3%)	
EF in % (mean±SD)	20 (6.9%)	45.5±11.6	46.8±11.8	44.4±11.4	0.094
Number of vessels (mean±SD)	0 (0%)	3.3±0.8	3.3±0.8	3.3±0.8	0.343
Procedure Status, n (%)	0 (0%)				0.187
Elective		251 (86.9%)	110 (84.0%)	141 (89.2%)	
Emergent		38 (13.1%)	21 (16.0%)	17 (10.8%)	
CPB time in mins (mean±SD)	0 (0%)	115.9±34.9	116.4±35.8	115.4±34.3	0.801
Cross clamp time in mins (mean±SD)	2 (0.7%)	72.0±24.7	73.1±24.6	71.1±24.7	0.383
Duration of mechanical ventilation in days (mean±SD)	6 (2.1%)	1.9±2.7	2.0±3.6	1.8±1.6	0.853
Length of stay in ICU in days (mean±SD)	1 (0.3%)	4.0±3.5	4.2±3.8	3.9±3.4	0.400
Length of stay in hospital in days (mean±SD)	0 (0%)	13.6±11.2	13.2±11.1	13.9±11.2	0.696

Postoperative complication

**Figure 1:**
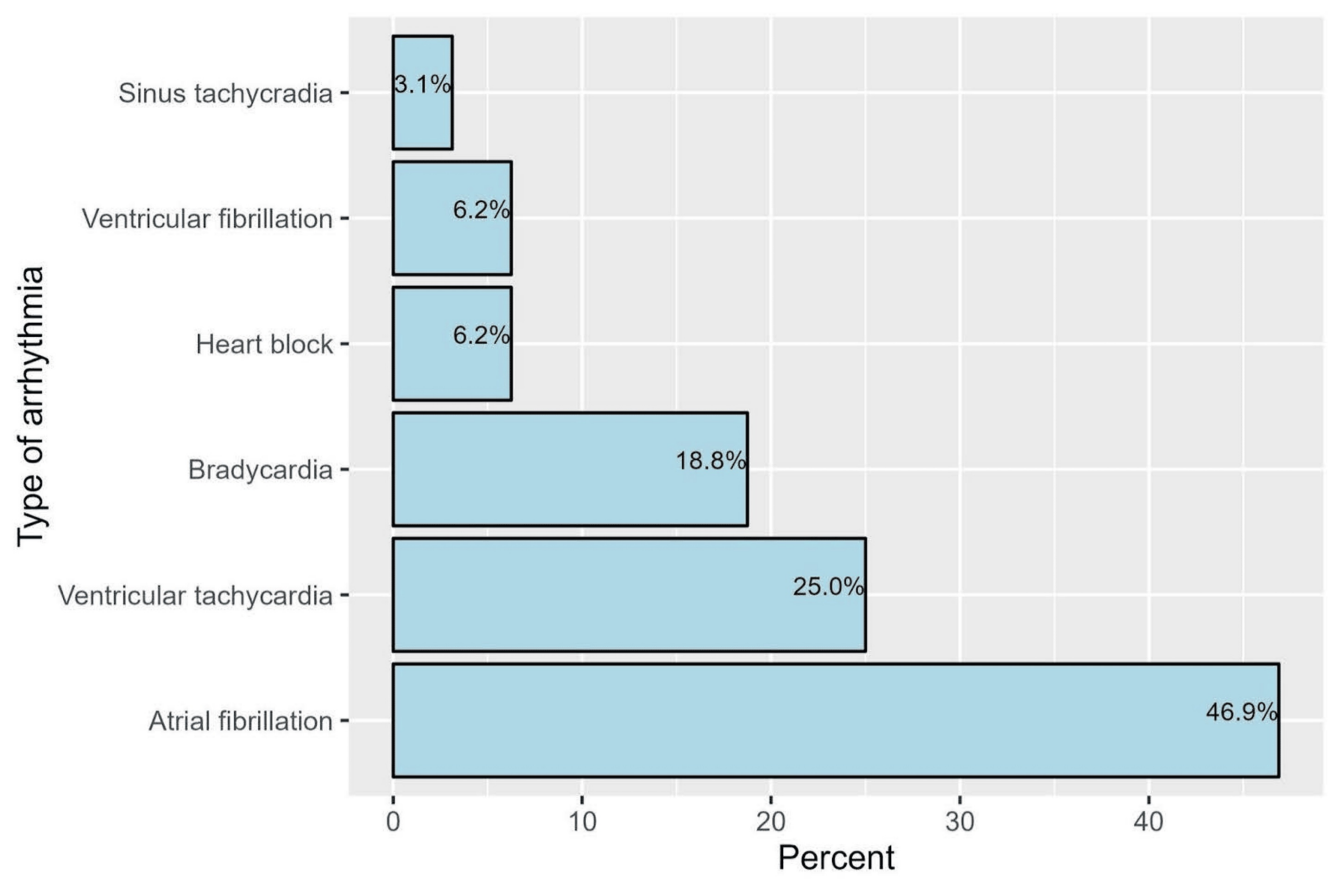
The incidence of different types of postoperative arrhythmia Bar chart showing the distribution of the types of arrhythmia that patients developed postoperatively after undergoing on-pump CABG with atrial fibrillation being the most common at 46.9% followed by ventricular tachycardia at 25%, bradycardia at 18.8%, heart block at 6.2%, ventricular fibrillation at 6.2%, and the least was sinus tachycardia at 3.1% CABG, coronary artery bypass grafting

Among patients who underwent CABG, the rates of specific complications were as follows: postoperative pneumonia was observed in 2.4% of patients, postoperative urinary tract infection occurred in 1.7%, postoperative wound infections were present in 6.9%, and the need for postoperative blood transfusion was required by 18.3% of patients. 3.1% of patients were admitted for acute coronary syndrome within 30 days postsurgery, and an additional 1.7% were admitted within 365 days. Low cardiac output syndrome occurred in 7.3% of cases, while 3.5% experienced a stroke after discharge within 30 days. Arrhythmia was noted in 11.1% of patients, of whom the most common types of arrhythmias were atrial fibrillation (46.9%) and ventricular tachycardia (25.0%) (Figure 1). 4.2% required pacemaker insertion. Postoperative stroke occurred in 12 patients with the majority being from an ischemic origin (83.3%) as indicated by the postoperative computed tomography with the rest being hemorrhagic (16.7%) (Table 3).

**Table 3:** Postoperative complication Fisher's exact test or Pearson's Chi-squared test was used for the categorical variables. *Infections include postoperative pneumonia, UTI, or wound infection UTI: urinary tract infection, postop Hb: postoperative hemoglobin, ACS: acute coronary syndrome, HF: heart failure

Characteristic	Missing	Overall, N=289	HbA1C <7%, N=131	HbA1C >7%, N=158	p-value
pneumonia, n (%)	0 (0%)				0.705
No		282 (97.6%)	127 (45.0%)	155 (55.0%)	
Yes		7 (2.4%)	4 (57.1%)	3 (42.9%)	
UTI, n (%)	0 (0%)				0.662
No		284 (98.3%)	128 (45.1%)	156 (54.9%)	
Yes		5 (1.7%)	3 (60.0%)	2 (40.0%)	
Wound infection, n (%)	0 (0%)				0.976
No		269 (93.1%)	122 (45.4%)	147 (54.6%)	
Yes		20 (6.9%)	9 (45.0%)	11 (55.0%)	
Any infection*, n (%)	0 (0%)				0.601
No		261 (90.3%)	117 (44.8%)	144 (55.2%)	
Yes		28 (9.7%)	14 (50.0%)	14 (50.0%)	
Need for postoperative blood transfusion, n (%)	0 (0%)				0.766
No		236 (81.7%)	106 (44.9%)	130 (55.1%)	
Yes		53 (18.3%)	25 (47.2%)	28 (52.8%)	
Postop Hb (day 1) in g/dL (mean±SD)	1 (0.3%)	9.2±1.4	9.4±1.7	9.0±1.1	0.023
Admission for ACS - within 30 days from surgery, n (%)	0 (0%)				0.518
No		280 (96.9%)	128 (45.7%)	152 (54.3%)	
Yes		9 (3.1%)	3 (33.3%)	6 (66.7%)	
Admission for ACS - within 365 days from surgery, n (%)	0 (0%)				0.662
No		284 (98.3%)	128 (45.1%)	156 (54.9%)	
Yes		5 (1.7%)	3 (60.0%)	2 (40.0%)	
Mortality - within 30 days from surgery, n (%)	0 (0%)				0.938
No		271 (93.8%)	123 (45.4%)	148 (54.6%)	
Yes		18 (6.2%)	8 (44.4%)	10 (55.6%)	
Mortality - within 365 days from surgery, n (%)	0 (0%)				0.254
No		286 (99.0%)	131 (45.8%)	155 (54.2%)	
Yes		3 (1.0%)	0 (0.0%)	3 (100.0%)	
HF (low cardiac output syndrome), n (%)	0 (0%)				0.813
No		268 (92.7%)	122 (45.5%)	146 (54.5%)	
Yes		21 (7.3%)	9 (42.9%)	12 (57.1%)	
Admission for HF - within 30 days from surgery, n (%)	0 (0%)				0.705
No		282 (97.6%)	127 (45.0%)	155 (55.0%)	
Yes		7 (2.4%)	4 (57.1%)	3 (42.9%)	
Admission for HF - within 365 days from surgery, n (%)	0 (0%)				0.382
No		284 (98.3%)	130 (45.8%)	154 (54.2%)	
Yes		5 (1.7%)	1 (20.0%)	4 (80.0%)	
Stroke after discharge - within 30 days from surgery, n (%)	0 (0%)				0.520
No		279 (96.5%)	125 (44.8%)	154 (55.2%)	
Yes		10 (3.5%)	6 (60.0%)	4 (40.0%)	
Stroke after discharge - within 365 from surgery, n (%)	0 (0%)				>0.999
No		289 (100.0%)	131 (45.3%)	158 (54.7%)	
Yes		0 (0.0%)	0 (NA%)	0 (NA%)	
Arrhythmia, n (%)	0 (0%)				0.345
No		257 (88.9%)	119 (46.3%)	138 (53.7%)	
Yes		32 (11.1%)	12 (37.5%)	20 (62.5%)	
Need for pacemaker insertion, n (%)	0 (0%)				0.148
No		277 (95.8%)	128 (46.2%)	149 (53.8%)	
Yes		12 (4.2%)	3 (25.0%)	9 (75.0%)	
Stroke, n (%)	277 (96%)				>0.999
Hemorrhagic		2 (16.7%)	1 (50.0%)	1 (50.0%)	
Ischemic		10 (83.3%)	5 (50.0%)	5 (50.0%)	

Patients with uncontrolled levels of HbA1c had lower levels of postoperative Hb at postoperative day 1 with a mean Hb of 9.0 g/dL when compared to patients with controlled HbA1c levels (9.4 g/dL, p=0.023). No other significant associations were observed between the controlled and uncontrolled HbA1c groups (Table 3).

**Figure 2:**
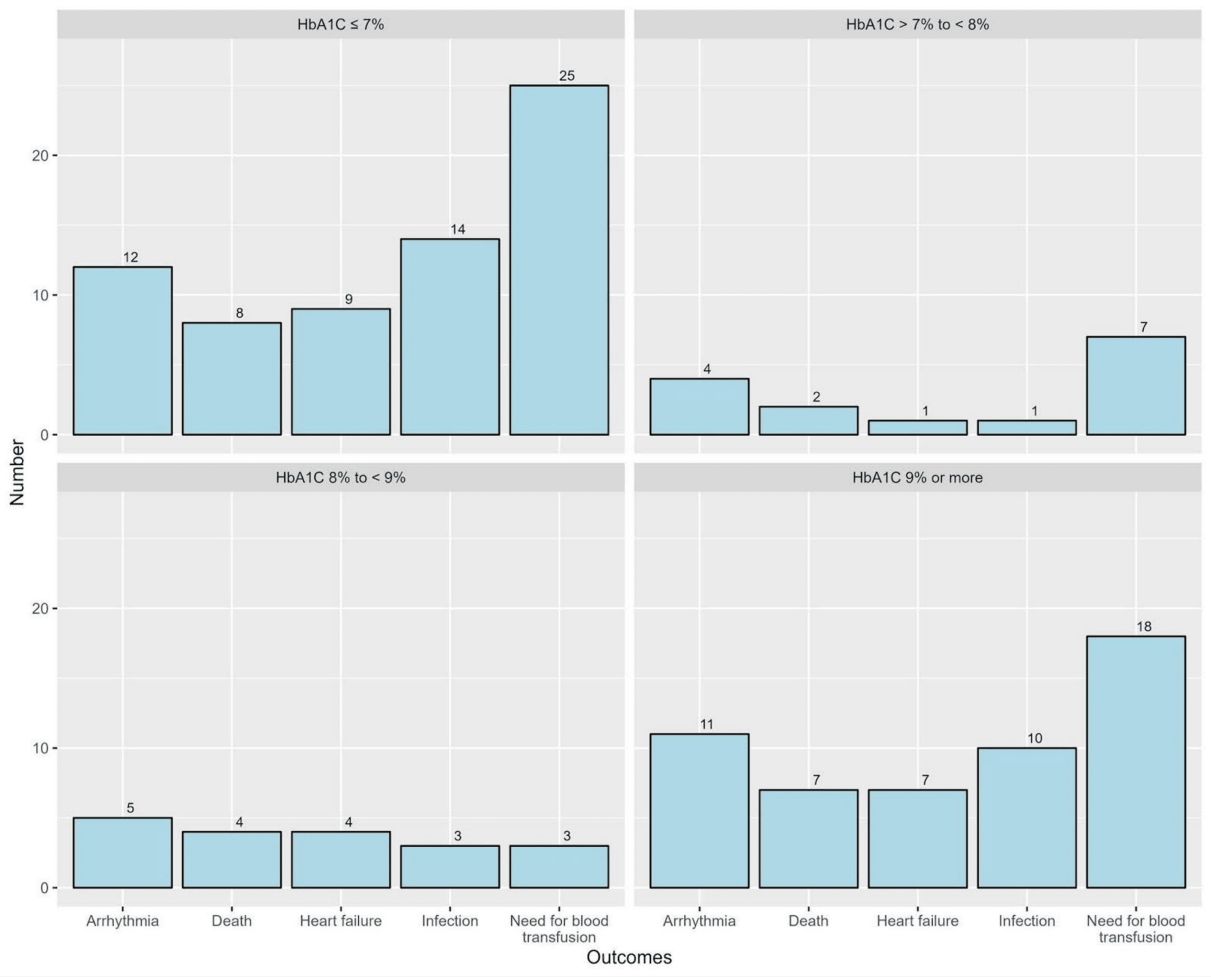
Frequencies of complications across different levels of HbA1C Bar chart depicting and comparing the frequencies of postoperative complications at different levels of HbA1C control. In the HbA1C <7%, HbA1C >7% to <8%, and HbA1C >9% groups, the incidence of the need for blood transfusion products was the most prevalent postoperative complication at a rate of 25, 7, and 18, respectively. In the 8% to <9% group, the most common complication was the development of postoperative arrhythmia with five patients developing the complication. HbA1c, glycosylated hemoglobin

Across different levels of HbA1C, there were no significant differences in the incidence of complications (p=0.788 for infection, p=0.372 for the need for blood transfusion, p=0.721 for heart failure, p=0.692 for arrhythmia, and p=0.712 for mortality, Figure 2).

Logistical regression analysis for predictors of postoperative complications

**Figure 3:**
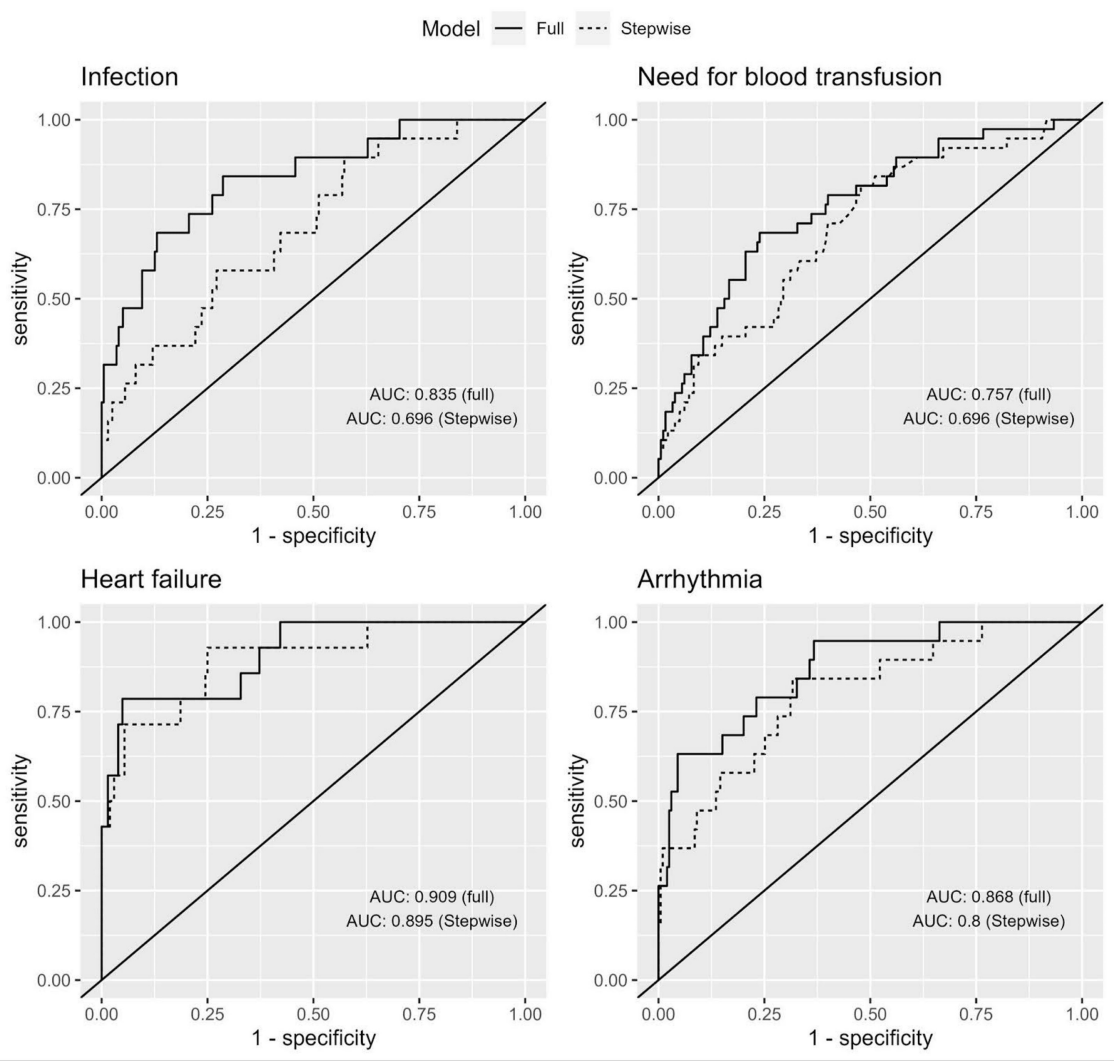
ROC curves for the logistic regression models (full and stepwise) for selected complications ROC: receiver operator characteristics, AUC: area under the curve

We constructed a series of multivariable logistic regression models to assess the risk factors for postoperative complications (each in a separate model). However, the models of the following outcomes were not fit to the data because the dependent variable occurred in a small number of patients: acute coronary syndrome within 30 and 365 days of surgery and stroke within 30 and 365 days of surgery. In the respective tables, we demonstrated the results of the univariable analysis as well as models containing all the independent variables (full models) and models with the retained independent variables after backward selection (stepwise models). Receiver operator characteristics (ROC) curves that compare the area under the curve (AUC) of full and stepwise models are demonstrated in Figure 3. For the development of infection, the AUC was 0.835 (95% CI, 0.736 to 0.936) for the full model and 0.696 (95% CI, 0.576 to 0.817) for the stepwise model. Similarly, for the need for blood transfusion, the AUC was 0.757 (95% CI, 0.673 to 0.842) for the full model and 0.696 (95% CI, 0.606 to 0.786) for the stepwise model. For heart failure, the AUC was 0.909 (95% CI, 0.826 to 0.992) for the full model and 0.895 (95% CI, 0.801 to 0.990) for the stepwise model. Finally, for arrhythmia, the AUC was 0.868 (95% CI, 0.782 to 0.953) for the full model and 0.800 (95% CI, 0.692 to 0.908) for the stepwise model. Below, we demonstrated the results of the stepwise models. The AUC values of the stepwise regression models demonstrated comparable predictive performance compared to those of the full models across all four outcomes.

For the risk of infection, patients with COPD (OR=27.5, 95% CI 0.98 to 777, p=0.027) and increased length of stay in the ICU were linked to a higher risk of infection (OR=1.14, 95% CI 1.03 to 1.28, p=0.017, Table 4). 

**Table 4:** Analysis of predictors of postoperative infection *Statistical significance COPD: chronic obstructive pulmonary disease

Characteristic	Univariate			Full			Stepwise		
	OR	95% CI	p-value	OR	95% CI	p-value	OR	95% CI	p-value
Age	0.97	0.93, 1.01	0.128	0.96	0.89, 1.03	0.229	0.97	0.92, 1.01	0.134
COPD									
No	Ref	Ref		Ref	Ref		Ref	Ref	
Yes	9.63	0.37, 248	0.113	10.6	0.22, 561	0.208	27.5	0.98, 777	0.027^*^
Cross clamp time	1.00	0.98, 1.01	0.798	0.97	0.92, 1.01	0.191	0.98	0.95, 1.00	0.054^*^

As for the need for postoperative blood transfusion, the status of the surgical procedure played a significant role, with emergent procedures showing a considerably higher risk of the need for transfusion compared to elective ones (OR=4.00, 95% CI 1.36 to 11.5, p=0.010) (Table 5).

**Table 5:** Analysis of predictors of postoperative need for blood transfusion *Statistical significance CPB: cardiopulmonary bypass, ICU: intensive care unit

Characteristic	Univariate			Full			Stepwise		
	OR	95% CI	p-value	OR	95% CI	p-value	OR	95% CI	p-value
Smoking									
No	Ref	Ref		Ref	Ref		Ref	Ref	
Yes	0.38	0.13, 0.94	0.055	0.40	0.10, 1.24	0.139	0.41	0.11, 1.13	0.113
Procedure status									
Elective	Ref	Ref		Ref	Ref		Ref	Ref	
Emergent	2.74	1.27, 5.75	0.008	4.00	1.36, 11.5	0.010	3.32	1.28, 8.33	0.011*
CPB time	1.00	0.99, 1.01	0.906	1.01	0.98, 1.03	0.568			
Cross clamp time	1.00	0.99, 1.01	0.925	0.99	0.96, 1.02	0.488			
Duration of mechanical ventilation	1.11	1.01, 1.27	0.059	1.19	0.94, 1.58	0.182	1.20	0.98, 1.56	0.103
Length of stay in ICU	1.05	0.98, 1.13	0.153	0.85	0.63, 1.07	0.206	0.86	0.66, 1.03	0.167
Length of stay in hospital	1.03	1.00, 1.05	0.022	1.04	1.00, 1.08	0.060	1.03	1.00, 1.06	0.062

Elevated WBC count, systemic atherosclerosis, and dialysis were significantly associated with an increased risk of postoperative low cardiac output syndrome (OR=1.30, 95% CI 1.03 to 1.70, p=0.041) (Table 6).

**Table 6:** Analysis of predictors of low cardiac output syndrome *Statistical significance PVD: peripheral vascular disease, COPD: chronic obstructive pulmonary disease, SBP: systolic blood pressure, DBP: diastolic blood pressure, HR: heart rate, WBC: white blood cells, CPB: cardiopulmonary bypass, HbA1C: glycosylated hemoglobin A1

Characteristic	Univariate						Full				Stepwise		
	OR	95% CI		p-value		OR		95% CI	p-value	OR		95% CI	p-value
PVD													
No	Ref	Ref				Ref		Ref		Ref		Ref	
Yes	28.1	2.58, 621		0.007		14.2		0.35, 1,010	0.170	21.3		1.00, 684	0.045*
Dialysis													
No	Ref	Ref				Ref		Ref		Ref		Ref	
Yes	3.30	0.16, 23.7		0.296		24.0		0.01, 48,233	0.383	34.9		0.99, 959	0.029*
COPD													
No	Ref	Ref				Ref		Ref		Ref		Ref	
Yes	13.4	0.52, 346		0.071		NA		0.00, NA	0.994	NA		0.00, NA	0.994
Stroke													
No	Ref	Ref				Ref		Ref		Ref		Ref	
Yes	1.17	0.06, 6.48		0.884		0.00		NA	0.993	0.00			0.993
SBP	1.01	1.00, 1.03		0.135		1.04		0.99, 1.10	0.126	1.03		1.00, 1.07	0.064
DBP	1.01	0.98, 1.04		0.552		0.99		0.91, 1.07	0.743				
WBC	1.01	0.97, 1.03		0.527		1.31		0.97, 1.80	0.082	1.30		1.03, 1.70	0.041*
CPB time	1.01	0.99, 1.02		0.278		1.01		0.96, 1.06	0.578	1.01		1.00, 1.03	0.117
HbA1C													
Controlled (HbA1C<7%)	Ref	Ref				Ref		Ref		Ref		Ref	
Uncontrolled (HbA1C>7%)	1.11	0.46, 2.81		0.813		4.74		0.49, 97.8	0.242	3.25		0.72, 20.1	0.153

Elevated SBP (OR=1.04, 95% CI 1.01 to 1.08, p=0.025), higher creatinine levels (OR=1.01, 95% CI 1.00 to 1.02, p=0.029), and increased length of hospital stay (OR=1.03, 95% CI 1.00 to 1.05, p=0.025) were found to be significant risk factors of postoperative arrhythmia (Table 7).

**Table 7:** Analysis of predictors of postoperative arrhythmia *Statistical significance COPD: chronic obstructive pulmonary disease, SBP: systolic blood pressure, CKD: chronic kidney disease

Characteristic	Univariate			Full			Stepwise		
	OR	95% CI	p-value	OR	95% CI	p-value	OR	95% CI	p-value	
CKD										
No	Ref	Ref		Ref	Ref		Ref	Ref		
Yes	1.08	0.16, 4.07	0.925	0.01	0.00, 1.05	0.125	0.06	0.00, 1.52	0.199	
Dialysis										
No	Ref	Ref		Ref	Ref		Ref	Ref		
Yes	0.00		0.989	0.00		0.995	0.00	NA	0.995	
COPD										
No	Ref	Ref		Ref	Ref		Ref	Ref		
Yes	8.26	0.32, 212	0.139	NA	0.00, NA	0.993	NA	0.00, NA	0.994	
Stroke										
No	Ref	Ref		Ref	Ref		Ref	Ref		
Yes	0.00		0.989	0.00		0.992	0.00		0.993	
SBP	1.02	1.00, 1.03	0.035	1.04	1.01, 1.08	0.025	1.03	1.01, 1.06	0.005*	
Creatinine	1.00	1.00, 1.00	0.229	1.01	1.00, 1.03	0.091	1.01	1.00, 1.02	0.029*	

Survival analysis

**Figure 4:**
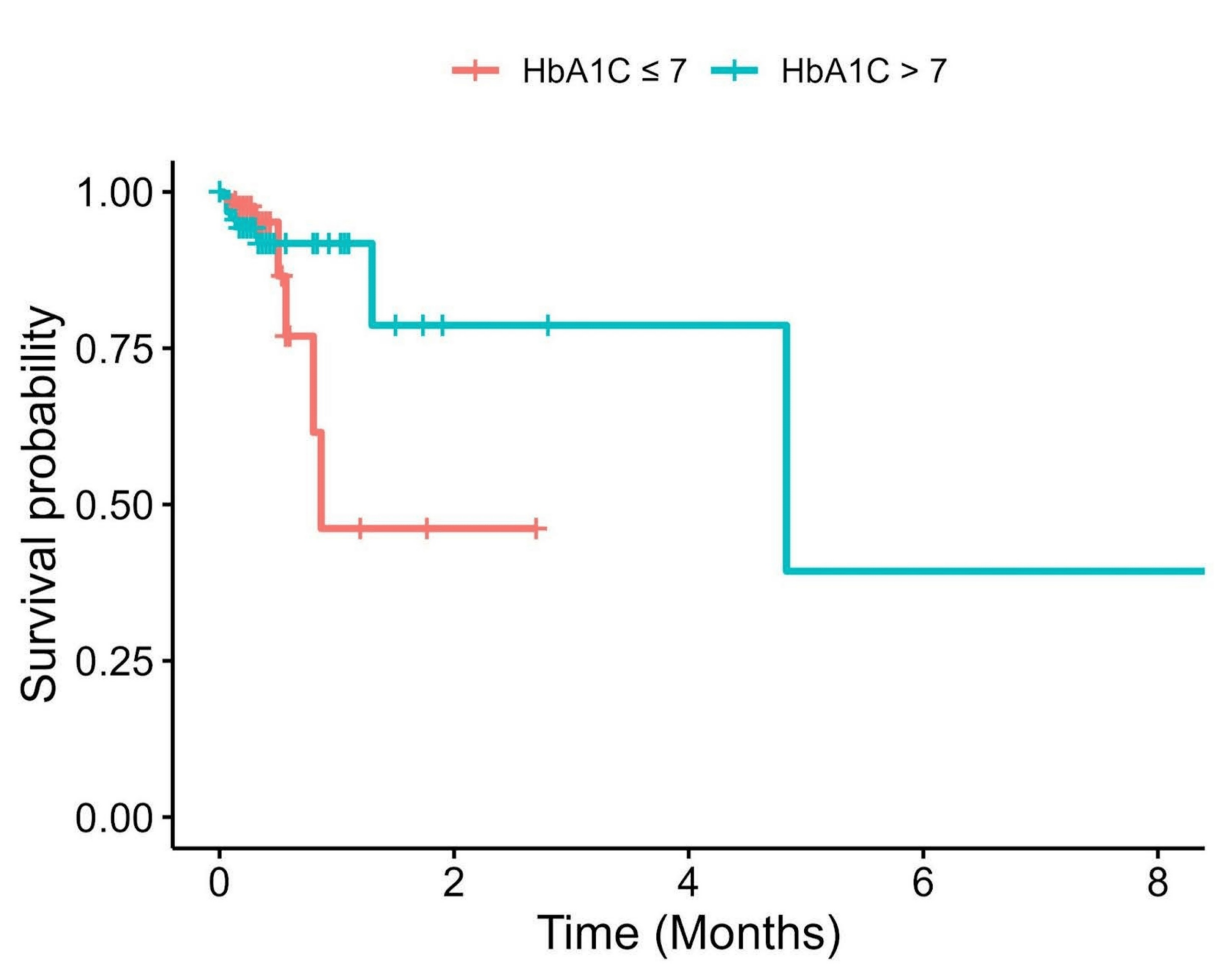
Kaplan-Meier curve depicting the survival of patients with controlled and uncontrolled levels of HbA1C There was no significant difference in survival between the two groups of patients HbA1C, glycosylated hemoglobin

In our cohort, 21 (7.2%) patients passed away. Mortality within 30 days postsurgery was observed in 18 (6.2%) patients, while three (1.0%) experienced mortality within 365 days. There was no significant difference in survival between patients when comparing controlled and uncontrolled HbA1c levels (p log-rank test=0.993, Figure 4). Based on the bivariate Cox proportional hazard analysis, we incorporated the variables with p-values <0.2; these included smoking, HR, Hb concentration, creatinine, troponin, number of vessels, procedure status, CPB time, cross-clamp time, duration of mechanical ventilation, and the length of hospital stay. 

The multivariable hazards regression analysis showed that increased risk of mortality was associated with smoking (HR=6.04, 95% CI, 1.23 to 29.6, p=0.026) and undergoing emergent surgery (HR=13.40, 95% CI, 3.09 to 58.1, p<0.001) (Table 8).

**Table 8:** Regression analysis for the hazard of mortality within 30 days *Statistical significance HR: heart rate, Hb: hemoglobin, CPB: cardiopulmonary bypass

Characteristic	Univariable			Multivariable		
	HR	95% CI	p-value	HR	95% CI	p-value
Smoking						
No	Ref	Ref		Ref	Ref	
Yes	2.31	0.89, 5.99	0.085	6.04	1.23, 29.6	0.026*
HR	1.02	1.00, 1.04	0.052	1.01	0.98, 1.05	0.453
Creatinine	1.00	1.00, 1.00	0.007	1.00	1.00, 1.00	0.144
Procedure status						
Elective	Ref	Ref		Ref	Ref	
Emergent	7.36	2.97, 18.2	<0.001	13.4	3.09, 58.1	<0.001*
CPB time	1.01	1.00, 1.02	0.005	1.02	0.99, 1.05	0.136
Cross clamp time	1.02	1.00, 1.03	0.018	0.99	0.95, 1.02	0.465
Duration of mechanical ventilation	1.06	1.01, 1.12	0.020	1.10	0.96, 1.26	0.162

## Discussion

In our study, we assessed 289 adult patients who underwent on-pump CABG and we looked at the effects of elevated HbA1c on this population and assessed if HbA1c can be used as a predictive biological marker for postoperative complications and major adverse cardiac events. It was found that in our population group, elevated preoperative HbA1c levels (>7%) did not correlate to an increased risk of developing complications. 

Chronic hyperglycemia, as indicated by an elevated HbA1C level, has been demonstrated to exacerbate cell damage. It is also believed to hinder endothelial function, contribute to oxidative stress and inflammation, reduce leukocyte activity, and delay recovery. In addition, acute glucose spikes also cause oxidative stress and endothelial dysfunction [11-15]. 

Multiple studies have been performed to assess the use of HbA1c as a predictor of major adverse cardiac events with a variety of results. On one hand, it has been stated that elevated levels of HbA1c have been associated with increased levels of complications, with other studies contradicting the previously mentioned [16-21].

In a study performed by Halkos et al., they assessed 3089 patients who underwent CABG and had an HbA1c result preoperatively. An elevated HbA1c level was a predictor for in-hospital mortality (odds ratio: 1.40, P=0.019). ROC curve analysis revealed that HbA1c greater than 8.6% was associated with a four-fold increase in mortality. For each unit increase in HbA1c, there was a significantly increased risk of myocardial infarction (P<0.001), deep sternal wound infection (P<0.001), increased hospital stay (P<0.001), and increased risk of postoperative renal failure (P<0.001) [22].

In Robich et al. (2019), 6,415 patients underwent on-pump CABG and they grouped those patients into four groups: HbA1c less than 5.7% (n=1,713), 5.7% to 6.4% (n=2,505), 6.5% to 8.0% (n=1,377), and more than 8% (n=820). After adjustment for patients' risk, greater HbA1c values were not associated with higher rates of in-hospital death or morbidity. The risk of mortality, however, increased by 13% for every unit increase in HbA1c (adjusted hazard ratio, 1.13; 95% CI, 1.07 to 1.19; p<0.001), while other complications postoperatively after adjustment did not associate with HbA1c levels [19]. 

In contrast to the studies mentioned, other studies did not find an association between HbA1c levels and the development of mortality and morbidity [8-10,23]. 

In a meta-analysis performed by Chen et al., they reviewed 23 cohort studies and it was found that there is a reduced incidence of surgical site infections (OR=2:94, 95% CI 2.18-3.98), renal failure events (OR=1:63, 95% CI 1.13-2.33), and myocardial infarction events (OR=1:69, 95% CI 1.16-2.47) in patients with lower preoperative HbA1c levels. However, the higher level of preoperative HbA1c had no effect on the incidence of mortality or other adverse events [24].

In a study by Knapik et al., they assessed the effects of elevated HbA1c on 2,665 patients undergoing on-pump CABG with elevated HbA1c levels, increasing only the frequency of perioperative myocardial infarction (p=0.01) and did not have any effect on other postoperative complications, but there is a metanalysis performed by Corazzari et al. that contradicts the finding that HbA1c is associated with an increased risk of perioperative myocardial infarction [8,16].

A retrospective study of 893 patients by Tsuruta et al. assessed the use of HbA1c as a long-term predictor of complication with the results of the Kaplan-Meier’s survival showing no significant differences in all-cause or cardiac mortality (log-rank test, p=0.26, p=0.17, respectively) in patients with elevated HbA1c [9].

Multiple local studies assessing the effects of elevated HbA1c on complications agree with our findings. One study assessed the use of HbA1c as a predictor of prolonged in-hospital stay in patients who underwent on-pump CABG. They found that HbA1c was not associated with an increased hospital stay (P=0.367). The other study assessed if it can be used to predict the development of postoperative wound infection (sternal or leg wounds), and it was not found to have an association (P=0.830) [25,26].

The lack of a mortality difference between individuals with and without diabetes may potentially have been impacted by changes in practice over the last 10 years when compared to previously published studies, in particular, the consistent application of the left internal thoracic artery in CABG and enhancements to perioperative care: 1) critical care and anesthesia and 2) improved coronary artery disease medical therapy including antiplatelets, statins, and preoperative glucose control and adjustment with standardized guidelines and treatment with insulin and oral antidiabetic drugs. They could have all worked together to enhance the results for diabetic individuals undergoing CABG. When contrasted with recent reports, these distinctions might render the interpretation of earlier studies less trustworthy [27,28].

In our institute, we use a multidisciplinary approach to care for our patients throughout their surgical journey from the preoperative period to outpatient follow-up. Endocrinology referral is routine practice if patients are planning for elective surgery to adjust their glycemic status prior to operation. Intraoperatively, anesthesia management with insulin is used to optimize intraoperative glycemic control, and postoperatively intensive critical care management to avoid rapid glycemic spikes. This may explain our results that elevated levels of HbA1c were not found to be a predictor of compilation, as our comprehensive management of glycemic parameters may mask the effect of chronic hyperglycemia. 

In summary, the use of HbA1c as a predictor of postoperative complications has conflicting results in the literature. Some cohort studies showed that there is an association with others showing no effects and some showing that it can be used as a predictor of some complications. Multiple meta-analyses show similar conflicting results [16,24]. 

In our experience, high HbA1c results did not affect our postoperative outcomes so we recommend that surgery should proceed without delay, even if patients have elevated HbA1C levels. As for elective patients with low-risk features and anatomy, optimizing preoperative glycemic control can be considered. 

Limitations 

This study has certain limitations that need to be noted. First, errors in data extraction from health care electronic and medical records might occur in retrospective chart review studies. Second, due to missing, illegible, or ambiguous data, patients with paper files were not included. Third, it can be difficult to standardize the outcomes because our university hospital employs several cardiac surgeons. Fourth, the fact that this study was carried out in a tertiary hospital with staff endocrinologists may reflect our ability to control in-hospital glycemic levels. Fifth, HbA1c levels were not taken into account together with the values of fasting blood glucose. Sixth, we did not subgroup the patients to ascertain whether patients who were insulin dependent had worse results when compared to those whose diabetes was managed with diet or oral hypoglycemic agents. Seventh, the follow-up duration was only a year postoperatively, a longer follow-up duration is needed. Eighth, the majority of our population in the study were males, which may affect generalization to females. 

## Conclusions

Our study provides new data on the prevalence and clinical significance of preoperative HbA1c levels in the Saudi population. In our data, elevated preoperative HbA1c had no predictive value for early complications and intermediate postoperative outcomes. We recommend that even if patients have elevated levels we should go ahead with surgical coronary revascularization if the procedure is not elective. If the patient was undergoing an elective operation, they should be referred and evaluated by an endocrinologist to optimize glycemic control.
